# Association of Prolonged Breastfeeding With Early Childhood Caries Using Propensity Score Matching in the French Longitudinal Study of Children (ELFE Cohort)

**DOI:** 10.1111/cdoe.70012

**Published:** 2025-08-27

**Authors:** Untray Brown, Sylvie Azogui‐Levy, Cathy Nabet, Marie‐Noëlle Dufourg, Marie‐Aline Charles, Joséphine Kerguen, Monique Kaminski, Laetitia Marchand‐Martin, Alice Germa

**Affiliations:** ^1^ UMR 1153, CRESS, Obstetric, Perinatal, Paediatric Life Course Epidemiology, OPPaLE INSERM, INRAE, Université Paris Cité Paris France; ^2^ Faculty of Health, UFR Odontology Université Paris Cité Montrouge France; ^3^ UR 3412, Laboratory of Health Education and Promotion, LEPS Sorbonne Paris Nord University Bobigny Cedex France; ^4^ Faculty of Health, UFR Odontology Toulouse Université Toulouse France; ^5^ Department of Odontology, Toulouse University Hospital (CHU de Toulouse), Toulouse University Toulouse France; ^6^ UMR1295, Center for Epidemiology and Research in POPulation Health, CERPOP INSERM, Toulouse University Toulouse France; ^7^ Ined, Inserm Elfe Joint Unit Aubervilliers France; ^8^ Department of Odontology Charles Foix Hospital, AP‐HP Ivry sur Seine France

**Keywords:** birth cohort, breastfeeding, Early childhood caries, propensity score matching

## Abstract

**Objectives:**

The aim of this study was to investigate the link between prolonged breastfeeding (≥ 12 months) and early childhood caries (ECC) using propensity score matching (PSM) to account for observed confounders, reduce bias, and provide a more reliable estimate of this relationship.

**Methods:**

This study utilised data from the French Longitudinal Study of Children (ELFE Cohort), comprising 11 718 participants. PSM was employed to pair children who were breastfed for 12 months or longer with those breastfed for less than 12 months or not at all, controlling for shared risk factors such as socioeconomic status and dietary habits. Logistic regression models were conducted to examine the association between ECC, reported by the parents at 3.5 years, and prolonged breastfeeding.

**Results:**

Infants (7.6%) who were breastfed for 12 months or more exhibited twice the odds of developing ECC at 3.5 years compared to those breastfed for less than 12 months or not at all (OR = 2.20, 95% CI: 1.39, 3.47).

**Conclusion:**

Children breastfed for 12 or more months are at increased risk of developing ECC. Further research is needed to investigate specific breastfeeding practices that may contribute to this increased risk, with the aim of promoting prolonged breastfeeding while ensuring optimal oral health.

**Abbreviations**
aOR: adjusted OR
CCTIRS: Comite Consultatif sur le Traitement de l'Information en matière de Recherche dans le domaine de la Santé
CNIS: Conseil National de l'Information Statistique
CPP: Comité de Protection des Personnes
CRESS: Centre for Research in Epidemiology and Statistics
DAG: directed acyclic graph
ECC: early childhood caries
ELFE: French Longitudinal Study of Children
EPOPE: Obstetrical Perinatal and Pediatric Epidemiology Research Team
GEE: generalised estimating equations
INSERM: French National Institute of Health and Medical Research
M1: main analysis
MAR: missing at random
Matchets: nonparametric preprocessing for parametric causal inference
MatchThem: matching and weighting after multiple imputation
MICE: multiple imputation chained equations
*N*
: population
*n*
: sample population
NNT: number needed to treat
OR: odds ratio
PSM: propensity score matching
S1: sensitivity analysis 1
S2: sensitivity analysis 2
S3: sensitivity analysis 3
S4: sensitivity analysis 4
SMD: standardised mean difference

## 1 Introduction

Early childhood caries (ECC) is a significant global health challenge, affecting up to approximately 48% of children under 6 years old, based on data from 29 countries [1]. In France, the prevalence of ECC was estimated to be 16% at the age of 5–6 years [2]. ECC is a biofilm‐mediated, sugar‐driven, multifactorial, and dynamic disease characterised by alternating phases of demineralisation and remineralisation, affecting both primary and permanent dentitions throughout life [3]. Poor oral health in children imposes substantial societal burdens, with untreated ECC leading to severe complications such as chronic pain, infections, and nutritional deficiencies [4]. ECC is closely linked to socioeconomic factors, with a higher prevalence among disadvantaged populations [5, 6, 7]. Prolonged breastfeeding, defined as breastfeeding for 12 months or more, is also influenced by social, cultural, and economic factors, including breastfeeding norms, maternal employment and socioeconomic status [8, 9, 10, 11].

Both the World Health Organization (WHO) and the American Academy of Pediatrics (AAP) recommend exclusive breastfeeding for the first 6 months, followed by the introduction of appropriate complementary foods thereafter, up to 2 years of age or beyond [12, 13]. The American Academy of Pediatric Dentistry (AAPD) supports breastfeeding prior to 12 months and advises minimising added sugar exposure in the diets of children under 24 months of age to promote optimal health and development [14]. While breastfeeding until 6 months during infancy may offer protection against ECC [15], further research is needed to fully understand the potential heightened risk of caries in children breastfed beyond 12 months [16, 17]. Several studies employing diverse methodologies have explored the association between prolonged breastfeeding and ECC [18, 19, 20, 21]. ECC is associated with social and economic factors including poor oral hygiene, inappropriate infant feeding practices, and socioeconomic factors such as maternal education and household income [22].

While these studies provide valuable insights, variations in the consideration and retention of confounding factors between studies may affect association estimates. A gap exists in understanding breastfeeding beyond 12 months, with disparities likely due to shared and overlapping risk factors between breastfeeding and ECC. Given the impracticality of randomising breastfeeding conditions, this study aimed to investigate the association between breastfeeding beyond 12 months and the development of ECC, using the Propensity Score Matching (PSM). To address confounding factors [23] PSM approximated randomised conditions by matching individuals with similar characteristics in exposed and unexposed groups, minimising selection bias and enhancing comparability [24].

## 2 Methods

### 2.1 Setting

This study draws from the ELFE study, which is a French national birth cohort. The cohort's primary aim is to comprehensively investigate the determinants influencing children's development, health, and socialisation from birth through to adulthood, employing a multidisciplinary approach. Initiated in 2011, the cohort encompasses a diverse national sample of children continuously monitored since birth in France. Inclusion criteria required participants to be live births at 33 weeks' gestation or later, with mothers aged 18 years and above, and no intention to depart from France within the subsequent 3 years.

### 2.2 Study Design

A total of 349 maternity units were randomly selected in metropolitan France and the children were recruited at birth from the 320 maternity units that agreed to participate. Over 96% (*n* = 37 494) of eligible mothers were approached during their maternity stay, with 51% consenting to participate, ultimately resulting in a cohort of 18 329 babies [25]. Of the 18 329 babies initially identified, exclusions were made due to missing data on the exposure or outcome, resulting in a final study population of 11 718 infants.

### 2.3 Data Collection

Baseline data were collected from hospital records (type of delivery, maternal birth location, smoking prior to pregnancy, and prenatal care) and maternal interviews at birth, followed by phone interviews conducted at 2 months, 1 year, 2 years, and 3.5 years. Other covariates were mainly collected at 2 months via phone interviews and questionnaires, including maternal age, education, employment status, household income, presence of grandparents, and the number of children in the household. Dietary variables, such as fruit juice and sugary drink consumption, were assessed through a self‐administered questionnaire, completed monthly by the parents between 2 and 10 months.

### 2.4 Exposure

Breastfeeding duration was assessed through maternal reports collected via structured questionnaires and interviews at multiple time points: at birth, 2 months, and 1 year. While the 1‐year phone interview provided key data on breastfeeding practices, information on breastfeeding that continued beyond 12 months was obtained retrospectively during the 2‐year follow‐up interview. For children breastfed at 1 year but not at 2, mothers reported the age of breastfeeding cessation. Breastfeeding was defined as any instance of breast milk provision, regardless of other dietary components. In this study, prolonged breastfeeding—defined as breastfeeding for 12 months or more—was the primary exposure variable. Children were categorised based on whether they had been breastfed for 12 months or longer, compared to those who stopped breastfeeding before 12 months or were never breastfed.

### 2.5 Outcome

The primary outcome, ECC, was assessed via a parent‐reported questionnaire during a phone interview at 3.5 years. Parents were asked whether their child had ever had at least one dental carious lesion.

### 2.6 Covariates

**FIGURE 1:**
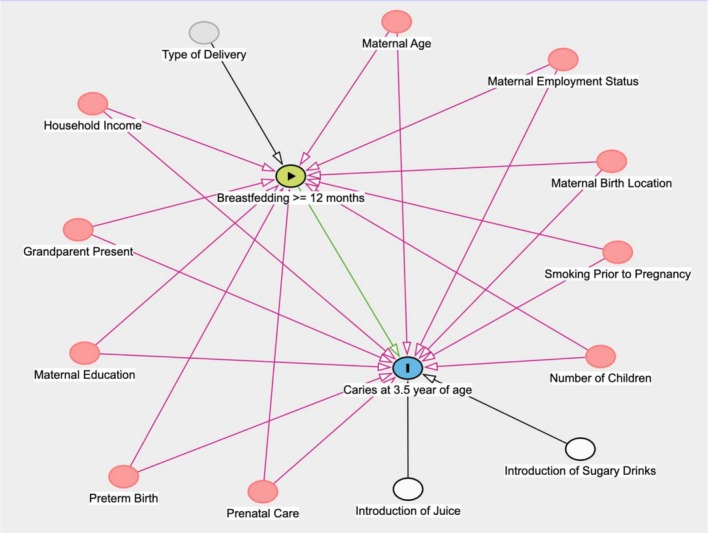
Directed acyclic graph of the relationship between prolonged breastfeeding and early childhood caries.

Covariates were selected based on prior literature and their relevance to both breastfeeding duration and ECC. A Directed Acyclic Graph (DAG) was developed to inform the identification of variables potentially associated with both the exposure and the outcome (Figure 1). The selected covariates included: maternal age [26], maternal education [27], maternal employment status [28], maternal birth location [29], presence of grandparent (grandmothers more often) [30], prenatal care (pregnancy information received) [27], smoking prior to pregnancy [31, 32], number of children [33], type of delivery [34], and preterm birth [35]. Household income was the income per unit of consumption of the household, categorised in quartiles [36]. Introduction of fruit juice and sugary drinks prior to 6 months was also considered as it is a risk factor for ECC. These variables were collected through medical records at birth and through maternal phone interviews and questionnaires at birth and 2 months postpartum.

### 2.7 Analysis Strategy

Descriptive analyses were conducted for each covariate to characterise the study population and examine differences between breastfeeding groups.

### 2.8 Propensity Score Matching

The propensity score (PS) was defined as each infant's probability of being breastfed for 12 months or more, estimated from a logistic regression model using observed covariates. The final study sample was compared to the entire initial cohort before exclusions of those lost to follow up or non‐respondents. PSM was employed. PSM with logistic regression is a widely used method in observational research for controlling confounding by observed variables [37, 38].

All listed covariates, with the exception of introduction of fruit juice and sugary drinks, were considered potential predictors of both breastfeeding and ECC and were included in the PS model regression.

PSM was implemented utilising MatchIt (Nonparametric preprocessing for Parametric Causal Inference) software in R [39]. PSM facilitated the balancing of observed covariates by matching prolonged breastfed and those less than 12 months or not at all according to measured maternal characteristics (maternal age, maternal birth location, education, employment status, household income) [40, 41]. A nearest neighbour 1–1 matching within a 0.2‐SD calliper without replacement was performed.

Infants breastfed for 12 months or more were matched with those breastfed for less than 12 months or not at all, based on similar PSs. After matching, the balance of covariates between groups was evaluated using standardised mean differences (SMD), with values less than 10% considered indicative of sufficient balance. The SMD is the difference in means or proportions divided by the pooled standard deviation. Matching quality was further assessed by examining whether key characteristics differed between matched infants and their counterparts. This process was repeated across multiple simulated datasets to evaluate the robustness of the matching.

### 2.9 Main Analysis

A reference odds ratio (OR) on the overall cohort was calculated using logistic regression prior to PSM. The baseline OR from this model provided an initial estimate of their association in the unadjusted model, serving as a reference point for the main and sensitivity analyses. Odds ratios were then calculated to quantify the association between prolonged breastfeeding exposure and ECC outcome using logistic regression analysis fitted by generalised estimating equations (GEE), which account for paired data [42, 43].

### 2.10 Sensitivity Analysis

First, logistic regression analyses were performed to calculate OR for the association of breastfeeding with ECC, adjusted for fruit juice and sugary drinks prior to 6 months. This adjustment ensured that the significant association between breastfeeding at 12 months and ECC at 3.5 years remained even after accounting for these dietary factors. Second and third, additional matching between infants breastfed 12 months or more and those breastfed less than 12 months or not breastfed at different ratios (1:2 and 1:3) was conducted to evaluate how changes in the matching ratio affected the estimated odds ratios and overall study findings. This explored the consistency of observed associations between breastfeeding duration and ECC risk across different scenarios.

### 2.11 Missing Data

For a sensitivity analysis, missing values on the ECC outcome were modelled and imputed using the Multiple Imputation Chained Equations (MICE) package in R [44]. Multiple imputation was performed using all previously described variables. A large number of predictors were included in order to make the assumption, missing at random, more plausible and ensure reliable imputed results. PS analysis was then performed on each imputed dataset to estimate the effect of exposure and estimates were pooled according to Rubin's rule, using the MatchThem package in R [45].

### 2.12 Weighting

A weighting procedure was applied to calculate the prevalence of ECC in the study population. To correct for non‐representativeness, statistical weights were calculated for each child based on the survey design, initial non‐response, and their 3.5‐year participation. This procedure ensured that the prevalence rates more accurately reflect the true occurrence of ECC in the entire population [25, 46]. Weights were not used for the matching.

## 3 Results

### 3.1 Study Population

**FIGURE 2:**
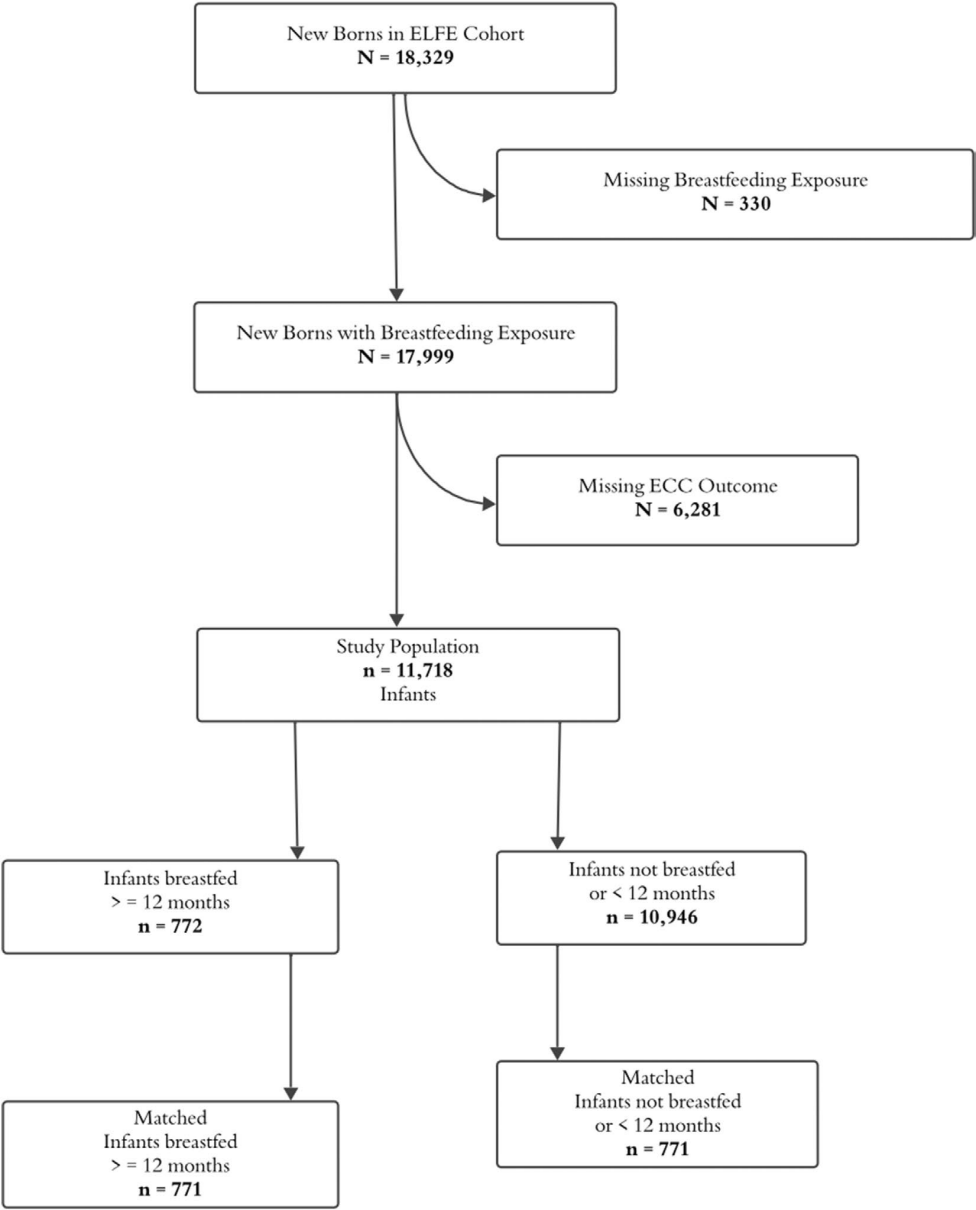
Study population.

**FIGURE 3:**
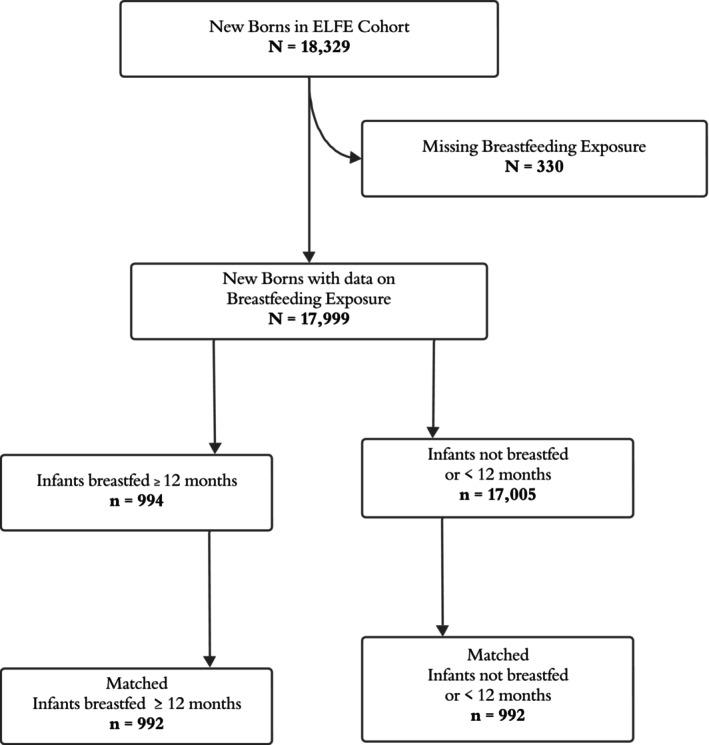
Study population after multiple imputation.

Out of 18 329 infants, those with data on breastfeeding at 12 months, excluding 330 (1.8%) with missing data, totalled 17 999. (Figure 2) Among these, 6281 (35%) had missing data on the ECC outcome, resulting in a study population of 11 718 infants with data on both breastfeeding exposure and ECC outcome. Out of the 11 718 infants included in the main analysis, 10 946 were non‐breastfed or breastfed for ≥ 12 months, and 772 were breastfed for 12 months or more. A comparison between the study population and the population with missing ECC data revealed differences in several demographic and socioeconomic characteristics. The study population tended to be older, more educated, and had higher employment and income levels compared to the group with missing data. They were also more likely to have been born in France, received prenatal care, and had a lower prevalence of smoking before pregnancy. In contrast, the group with missing data had a higher proportion of younger mothers, lower education and income levels, and a greater presence of grandparents in the home. In the study, the prevalence of breastfeeding at 12 months was 5.5%, while the weighted prevalence of ECC was estimated at 3.4% (Figure 3).

### 3.2 Main Results

Mothers in the prolonged breastfeeding group were more likely to be older (31–40 years), foreign‐born, have multiple children, hold a university degree, and come from lower‐income households (Table 1). In contrast, mothers who breastfed for a shorter duration or not at all were more often professionally active and had a higher prevalence of smoking before pregnancy. However, gestational age showed minimal differences between the groups. The main result of the study, based on the PSM, is that infants breastfed for 12 months or more are at a higher risk of ECC, OR = 2.20 and a 95% CI (1.39, 3.47), compared to those who were not breastfed or breastfed for ≥ 12 months (Table 2).

**TABLE 1:** Population baseline characteristics: propensity scores before and after matching.

Characteristics	Initial sample		Before matching	Matched infants	After matching
	BF ≥ 12 m, *N* = 772, *n* (%)	BF < 12 m, *N* = 10 946, *n* (%)	SMD	BF < 12 m, *N* = 771, *n* (%)	SMD
Maternal age (years)					
≤ 25	42 (5.4)	1069 (9.8)	4.4	40 (5.2)	0.2
26–30	200 (25.9)	3745 (34.2)	8.3	217 (28.1)	2.2
31–35	325 (42.1)	4066 (37.1)	5.0	315 (40.9)	1.2
36–40	162 (21.0)	1738 (15.9)	5.1	166 (21.5)	0.5
> 40	43 (5.6)	327 (3.0)	2.6	33 (4.3)	1.3
Maternal birth location					
France	591 (76.6)	9980 (91.2)	14.6	612 (79.4)	2.8
Outside France	181 (23.4)	966 (8.8)	14.6	159 (20.6)	2.8
Maternal education					
Less than high school	44 (5.7)	488 (4.5)	1.2	42 (5.5)	0.2
High school	158 (20.5)	3081 (28.2)	7.7	148 (19.2)	1.3
Two‐year college	143 (18.5)	2607 (23.8)	5.3	152 (19.7)	1.2
University	407 (52.7)	4590 (41.9)	10.8	408 (52.9)	0.2
Missing data	20 (2.6)	180 (1.6)	1.0	21 (2.7)	0.1
Maternal employment status					
Professionally active	482 (62.4)	8396 (76.7)	14.3	477 (61.9)	0.5
Unemployed	95 (12.3)	1094 (10.0)	2.3	87 (11.3)	1.0
Housewife	135 (17.5)	915 (8.4)	9.1	145 (18.8)	1.3
Student	40 (5.2)	360 (3.3)	1.9	41 (5.3)	0.1
Missing data	20 (2.6)	181 (1.6)	1.0	21 (2.7)	0.1
Household income					
Low income	214 (27.7)	1974 (18.0)	9.7	232 (30.1)	2.4
Lower‐middle income	165 (21.4)	2951 (27.0)	5.6	152 (19.7)	1.7
Upper‐middle income	207 (26.8)	3101 (28.3)	1.5	213 (27.6)	0.8
High income	143 (18.5)	2434 (22.3)	3.8	137 (17.8)	0.7
Missing data	43 (5.6)	486 (4.4)	1.2	37 (4.8)	0.8
Grandparent in home					
Yes	17 (2.2)	167 (1.5)	0.7	7 (0.9)	1.3
No	738 (95.6)	10 612 (97.0)	1.4	747 (96.9)	1.3
Missing data	17 (2.2)	167 (1.5)	0.5	17 (2.2)	0.0
Smoking before pregnancy					
Yes	211 (27.3)	4606 (42.1)	14.8	209 (27.1)	0.2
No	557 (72.2)	6252 (57.1)	15.1	561 (72.8)	0.6
Missing data	4 (0.5)	88 (0.8)	0.3	1 (0.1)	0.4
Prenatal care					
Yes	479 (62.0)	6865 (62.7)	0.7	469 (60.8)	1.2
No	287 (37.2)	3919 (35.8)	1.7	297 (38.5)	1.3
Missing data	6 (0.8)	162 (1.5)	0.7	5 (0.7)	0.1
Preterm birth					
Yes	62 (8.1)	1208 (11.0)	3.1	51 (6.6)	1.5
No	702 (90.9)	9566 (87.4)	3.5	714 (92.6)	1.7
Missing data	8 (1.0)	172 (1.6)	0.6	6 (0.8)	0.2
Type of birth					
Spontaneous vaginal	557 (72.2)	7327 (66.9)	5.3	575 (74.6)	2.4
Forceps/Spatulas/Vacuum	78 (10.1)	1346 (12.3)	2.2	68 (8.8)	1.3
Caesarean	116 (15.0)	1985 (18.1)	3.1	108 (14.0)	1.0
Missing data	21 (2.7)	288 (2.7)	0.0	20 (2.6)	0.1
Number of children					
Two	270 (35.0)	5040 (46.1)	11.1	281 (36.5)	1.5
Three or more	284 (36.8)	3919 (35.8)	1.0	270 (35.0)	1.8
One	210 (27.2)	1844 (16.8)	10.4	213 (27.6)	0.4
Missing data	8 (1.0)	143 (1.3)	0.3	7 (0.9)	0.1

*Note:* *n* (%), by column; mean.

Abbreviations: BF, breastfeeding; SMD. standardised mean difference.

**TABLE 2:** Summary effects: association of prolonged breastfeeding and early childhood caries.

Analysis	Sample	*N*	OR	95% CI	*p*
Reference	Initial	11 718	3.11	2.32, 4.15	< 0.001
	Matched	1542	2.20	1.39, 3.47	< 0.001
SA 1	Matched (adjusted)^a^	1538	2.16	1.36, 3.43	< 0.001
SA 2	Matched ratio 1:2	2308	2.63	1.78, 3.89	< 0.001
SA 3	Matched ratio 1:3	3052	3.09	2.14, 4.45	< 0.001
SA 4	After MI^b^	1984	1.98	1.24, 3.17	0.004

Abbreviations: CI, confidence interval; OR, odds ratio; SA, sensitivity analysis (1–3).

^a^ Adjusted for the introduction of fruit juice and sugary drinks before 6 months.

^b^ Multiple imputation.

### 3.3 Sensitivity Results

The sensitivity analysis adjusting for fruit juice and sugary drinks showed a slightly lower odds ratio (OR = 2.16, 95% CI: 1.36, 3.43) compared to the primary matched analysis (OR = 2.20). The matching ratio 1:2 the OR = 2.63 and 95% CI (1.78, 3.89) and for 1:3 OR = 3.09, 95% CI (2.14, 4.45). After multiple imputation of the 6281 missing data on the ECC outcome (35%), 992 infants breastfed 12 months or more were matched to 992 not breastfed at 12 months. The association estimated an OR of 1.98 (95% CI: 1.24, 3.17) (Table 2).

## 4 Discussion

Overall, the findings indicate that infants breastfed for 12 months or more have a significantly higher risk of experiencing ECC compared to those not breastfed or breastfed for less than 12 months. The association between breastfeeding duration and ECC was analysed using a PS approach to adjust for confounding factors and to control for baseline differences between exposure groups. This method helps reduce potential bias due to unmeasured confounders. Consistency in estimated odds ratios across different matching ratios further reinforces the robustness and reliability of the findings.

This study employed PSM within the French national birth cohort (ELFE). Participants were matched based on observed maternal characteristics—age, birth location, education, employment status, and household income—to balance the covariates across breastfeeding groups. This technique addresses methodological challenges common in observational studies and helps mitigate selection bias. While no definitive breastfeeding threshold can be pinpointed at which oral health is affected, studies often examine durations such as 12, 18 or 24 months. Among these, 12 months is a practical reference for both preventive strategies and research purposes, as it aligns with the timing of primary tooth eruption and early oral exposure.

The early introduction of sugary beverages—such as fruit juices, herbal teas, and syrups—before 6 months of age has been recently linked to ECC [47]. Infants breastfed for longer durations who were also exposed to these drinks earlier or in larger quantities may have a higher risk of ECC. Early introduction may not only reduce breastfeeding duration by decreasing interest but also serve as an indicator of higher sugar consumption later in childhood. Although this variable could not be included in the PS due to its complex and not fully understood relationship with breastfeeding duration, it was considered as a potential marker of early sugar exposure (Table 3).

**TABLE 3:** Comparaison of the study population and the children with missing data on ECC.

Characteristic	Study population (*N* = 11,718)^a^, *n* (%)	Missing caries data (*N* = 6281)^a^, *n* (%)	*p* ^b^
Maternal age (years)			
≤ 25	1111 (9.5)	1573 (25.0)	< 0.001
26–30	3945 (33.7)	2095 (33.4)	
31–35	4391 (37.5)	1607 (25.6)	
36–40	1900 (16.2)	810 (12.9)	
> 40	370 (3.1)	191 (3.0)	
Missing data	1 (0.01)	5 (0.1)	
Maternal birth location			
France	10 571 (90.2)	5055 (80.5)	< 0.001
Outside France	1147 (9.8)	1187 (18.9)	
Missing data	0 (0.0)	39 (0.6)	
Maternal education			
Less than high school	532 (4.5)	757 (12.0)	< 0.001
High school	3239 (27.6)	2175 (34.6)	
Two‐year college	2750 (23.5)	815 (13.0)	
University	4997 (42.6)	954 (15.2)	
Missing data	200 (1.8)	1580 (25.2)	
Maternal employment status			
Professionally active	8878 (75.8)	2695 (42.9)	< 0.001
Unemployed	1189 (10.1)	780 (12.4)	
Housewife	1050 (9.0)	1038 (16.5)	
Student	400 (3.4)	173 (2.8)	
Missing data	201 (1.7)	1595 (25.4)	
Household income			
Low income	2188 (18.7)	1786 (28.4)	< 0.001
Lower‐middle income	3116 (26.6)	1220 (19.4)	
Upper‐middle income	3308 (28.2)	743 (11.8)	
High income	2577 (22.0)	517 (8.3)	
Missing data	529 (4.5)	2015 (32.1)	
Grandparent in home			
Yes	184 (1.6)	204 (3.3)	< 0.001
No	11 350 (96.9)	4600 (73.2)	
Missing data	184 (1.5)	1477 (23.5)	
Smoking before pregnancy			
Yes	4817 (41.1)	2767 (44.0)	< 0.001
No	6809 (58.1)	3452 (55.0)	
Missing data	92 (0.8)	62 (1.0)	
Prenatal care			
Yes	7344 (62.7)	2443 (38.9)	< 0.001
No	4206 (35.9)	3690 (58.7)	
Missing data	168 (1.4)	148 (2.4)	
Premature birth			
Yes	1270 (10.9)	818 (13.0)	< 0.001
No	10 268 (87.6)	5353 (85.2)	
Missing data	180 (1.5)	110 (1.8)	
Type of birth			
Spontaneous vaginal	7884 (67.3)	4158 (66.2)	0.5
Forceps/Spatulas/Vacuum	1424 (12.2)	778 (12.4)	
Caesarean	2101 (17.9)	1165 (18.5)	
Missing data	309 (2.6)	180 (2.9)	
Number of children			
One	5310 (45.3)	2796 (44.5)	< 0.001
Two	4203 (35.9)	2003 (31.9)	
Three or more	2054 (17.5)	1382 (22.0)	
Missing data	151 (1.3)	100 (1.6)	

^a^ *n* (%), by column.

^b^ Pearson's chi‐squared test was used to calculate all *p*‐values.

Previous studies investigating the association between prolonged breastfeeding and ECC have reported mixed findings, partly due to methodological differences. For instance, cohort studies by Abanto et al. [18], van Meijeren‐van Lunteren et al. [20], Devenish et al. [21] and Peres et al. [48] utilised statistical approaches such as the G‐formula, negative hurdle binomial models, and marginal structural models, producing odds ratios ranging from 1.13 to 1.9. Building on this literature, the present study applied PSM combined with logistic regression, offering a more precise estimate of the association while adjusting for a broader range of cultural, social, and economic factors that influence both breastfeeding practices and ECC risk.

A notable strength of this study lies in its methodological advancement over earlier work. Unlike prior studies that used unmatched comparisons or basic regression models, this analysis accounted for complex confounding structures through PSM, resulting in well‐balanced comparison groups. This allowed for a more accurate estimation of ECC risk specifically attributable to prolonged breastfeeding, even beyond the 1‐year mark. Furthermore, the study emphasised the potential mediating role of sugary drink consumption, offering a nuanced perspective rarely addressed in previous research. This approach highlights that prolonged breastfeeding might delay the introduction of sugary drinks, a potential confounder in the relationship with ECC.

The methodological rigour of this study offers multiple contributions. PSM with logistic regression is a widely used approach in observational research for controlling confounding by observed variables. Given the large sample size and availability of extensive sociodemographic data, this method allowed for matching on a wide range of factors despite the relatively small number of children breastfed at 12 months. Although PSM cannot fully eliminate the impact of unmeasured confounding or model misspecification, the inclusion of multiple sensitivity analyses helped strengthen the robustness of the findings.

Regarding outcome measurement, ECC was based on maternal report rather than clinical examination, which may introduce some misclassification. While maternal‐reported data are less accurate than clinical assessments [49], they remain a practical and scalable method in large population‐based cohorts where clinical data collection is often not feasible. Moreover, ECC may have been underestimated, as parents are more likely to report only visibly cavitated lesions, while posterior lesions (e.g., on molars) may go unnoticed. Anterior teeth—commonly affected at age 3.5—are more visible and thus more likely to be reported. This may have resulted in non‐differential misclassification of the outcome. As a result, some children with ECC may have been misclassified as unaffected. However, there is no reason to believe that this underestimation of ECC would differ between the exposed and non‐exposed groups. Consequently, the observed association between prolonged breastfeeding and ECC may underestimate the true risk.

Despite this limitation, the findings are valuable, especially in the context of limited dental visits among young children in France, where the first dental visit often occurs around age 6, as part of a national prevention programme. In France, the estimated prevalence is 6% among children aged 5–6, though data on children under three are lacking [50]. Notably, when missing ECC data were imputed, the odds ratio for the association with prolonged breastfeeding was only slightly attenuated. This suggests that missing outcome data had minimal influence on the observed association.

The final sample sizes—1542 for complete cases and 1984 with imputed data—were sufficiently large to yield precise and reliable estimates, despite the sample size reduction due to unmatched cases. These findings align with and complement the results of Shrestha's 2024 systematic review, which identified prolonged and nocturnal breastfeeding as significant ECC risk factors [51]. While that review provided pooled estimates, the present study contributes a more detailed and contextually grounded analysis using advanced statistical techniques.

Ultimately, this study highlights the complexity of the relationship between breastfeeding duration and ECC. While breastfeeding provides numerous health benefits, the findings suggest that breastfeeding beyond 12 months—especially in the context of high sugar exposure or nocturnal feeding—may increase ECC risk. Given that nocturnal breastfeeding is more common than nocturnal bottle‐feeding [52], future research should further investigate specific practices, such as night‐time feeding, to inform targeted public health recommendations. These findings underscore the importance of balancing the promotion of breastfeeding with strategies to prevent ECC, supporting nuanced guidance that considers both nutritional and oral health outcomes.

## 5 Conclusion

Prolonged breastfeeding was associated with an increased risk of self‐reported ECC for infants breastfed for 12 or more months. Further research should weigh the strengths and limitations of PSM alongside regression and consider complementary methods or sensitivity analyses to enhance the robustness of the findings. Additionally, it is essential to explore strategies that support mothers while mitigating the impact of breastfeeding on their child's oral health.

## Ethics Statement

The ELFE study received approval from the national committee for statistical information (CNIS). Ethical clearance for data collection in maternity units and each follow‐up wave was obtained from the national advisory committee on information processing in health research (CCTIRS), the national data protection authority (CNIL), and, for invasive data collection such as biological sampling, the committee for protection of persons engaged in research (CPP).

## Consent

Recruitment and data collection began after obtaining informed consent from participating families, who planned to reside in metropolitan France for at least 3 years. Consent was given by both parents or the mother alone, with the father informed of his right to decline consent. Information and consent materials were available in French, Arabic, Turkish, and English, reflecting common languages among women giving birth in France.

## Conflicts of Interest

The authors declare no conflicts of interest.

## Data Availability

The EFLE studies follow a data‐sharing policy accessible at https://plateforme‐acces‐donnees‐elfe‐france.site.ined.fr/, with updates and further details on the ELFE website https://www.ELFE‐france.fr/en/. ELFE implements an open‐data policy, with an 18‐month exclusivity period for associated research teams. Study protocols, questionnaires, and the data catalogue are available online. Data access requests must be submitted through the platform for approval by the ELFE data‐access committee, following the ELFE data‐access policy downloadable from the platform.
